# Non-monotonic variation of flow strength in nanochannels grafted with end-charged polyelectrolyte layers†

**DOI:** 10.1039/d1ra06601c

**Published:** 2022-02-02

**Authors:** Peng Wu, Tao Sun, Xikai Jiang

**Affiliations:** College of Energy and Power Engineering, Inner Mongolia University of Technology Inner Mongolia Hohhot 010051 China wupeng@imut.edu.cn; China–EU Institute of Clean and Renewable Energy, Huazhong University of Science and Technology Wuhan Hubei 430074 China; State Key Laboratory of Nonlinear Mechanics, Institute of Mechanics, Chinese Academy of Sciences Beijing 100190 China

## Abstract

The electrokinetic transport of fluids, also called the electroosmotic flow (EOF), in micro/nanoscale devices occurs in promising applications such as electrokinetic energy conversion (EKEC) systems. Recently, EKEC systems grafted with end-charged polyelectrolyte (PE) layers (PELs) have been reported to exhibit higher efficiencies than those of intrinsic systems. Understanding the interplay between the end-charged PELs and electrical double layers (EDLs) on the EOF is crucial for designing highly efficient EKEC systems. The interplay between the end-charged PELs and EDLs on the strength of the EOF (*V*_0_) is studied by explicitly modeling the EOF through nanochannels grafted with end-charged PELs using atomic simulations. The variation of *V*_0_ is examined for nanochannels grafted with PELs at various separations (*d* = 3.5–0.4 nm) to cover various conformations of PEs, inlcuding mushroom, semi-dilute brushes, and concentrated brushes. We find that *V*_0_ follows a non-monotonic variation as *d* decreases and this is correlated with the conformation of the PEs. Specifically, as *d* decreases, *V*_0_ decreases first in the mushroom regime (*d* = 3.5–2.0 nm), and then *V*_0_ increases in the concentrated brush regime (*d* = 0.75–0.4 nm). Navigated by the continuum Navier–Stokes–Brinkman model, the above observations are rationalized by the competition between the driving effect from the spatial shift of ions in EDLs and the drag effect from PELs. The insights obtained in this work are important to guide the design of highly efficient EKEC systems by grafting end-charged PELs onto channel surfaces.

## Abstract 2

A non-monotonic variation of the strength of electroosmotic flow (*V*_0_) is reported for the electroosmotic flow through nanochannels grafted with end-charged polyelectrolytes at various separation using atomic simulations.

## Graphical abstract

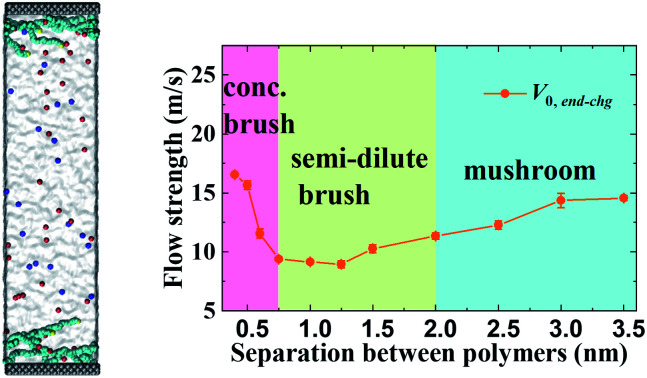

## 1 Introduction

The electrokinetic transport of fluids in micro/nanoscale devices has attracted increasing attention and has been widely used in applications ranging from the sensing and separation of molecules,^1^ the gating of liquids,^2^ the gating of ions,^3^ biomedical care^4^ to energy conversion systems.^5,6^ It has been acknowledged that the electroosmotic flow (EOF) can have a significant effect on the transport mechanism of molecules across nanopores.^7^ Electroosmosis can either compete or cooperate with electrophoresis in single molecular trapping in nanopores.^8^ Particular advances have been achieved in energy conversion systems based on electromechanical technologies to harvest energy from renewable sources,^9^ human motion^6,10^ or environmental waste heat.^11^ Electrokinetic energy conversion (EKEC) systems,^5^ which convert energy from micro/nanofluidic flow, have attracted increasing attention owing to their low maintenance and capability to provide a power source at the microscale. The early development of EKEC was pioneered by works from Kwok^12^ and Daiguji^13^ with an EKEC energy conversion efficiency of around 1%. With recent advances in nano-materials,^14^ nano-manufacturing^2^ and operation parameter optimization (*e.g.* adjusting temperature^11^), the EKEC systems have achieved significant progress. In a recent study, an efficiency of up to 50% was achieved for a ballistic electrostatic generator.^15^

The key ingredients for the working of EKEC systems include the electrical double layers (EDLs).^16^ As a charged or an ionizable substrate is immersed into the electrolyte solution, the charged surfaces attract counter-ions to balance the charge, resulting in the formation of EDLs at the interface of the electrolyte and substrate.^17,18^ With an external electric field tangential to the substrate, the ions in the EDLs move and transport the momentum to solvents, which generates the electrokinetic transport of fluids, resulting in electroosmotic flow (EOF).^17^ With the absence of the external electric field, the flow of electrolytes in nanochannels can be driven by external pressure differences. The transport of ions results in an electrical current, named the streaming current.^19^ The streaming current offers a simple and effective method to convert mechanical energy to electrical power.^12,13,15,20^ The electrokinetic transport of fluids plays a crucial role in EKEC devices, which justifies the need for fundamental research on electrokinetic transport.

As the driving force for the electrokinetic transport of fluids occurs at the interface between the substrate and electrolyte solution, the physical and chemical properties of the interface are critical for electrokinetic transport. Grafting polyelectrolyte (PE) brushes to the surfaces is a versatile method to modify the physical and chemical properties of interfaces,^21–23^ enabling applications such as ionic gates,^24^ single (bio)nanoparticle sensing,^25^ regulating ion transport,^26^ nanofluidic diodes,^27^ and current rectification.^28–30^ Moreover, the grafting properties of PE brushes may significantly affect the electrokinetic transport in the nanochannel. For example, the Donnan potential^31,32^ of the nanochannel significantly affects the electrokinetic transport in a biomimetic PE-modified nanochannel, which is modulated by the imposed gate voltages and the solution properties. The ion selectivity of the biomimetic nanopore and the preferential direction of the ionic current can be regulated by the grafting density of the PE brushes.^23,30^ The current rectification and ion concentration polarization effects are strongly affected by the grafting position of the PE brushes at the nanopore (on the inner or outer surfaces of the nanopore).^28^ Although the EOF in microchannels is significantly suppressed by neutral polymers,^33,34^ grafting polyelectrolyte layers (PELs) onto channel surfaces can be an effective way to enhance the charge density of channel surfaces and enhance the EOF velocity.^35^ Finally, channel surfaces grafted with charged PELs improve the performance of EKEC devices. Das and co-workers^36,37^ have reported that the streaming potential in soft channels (channels with PELs) is often larger than that in rigid channels and the efficiency of energy conversion of soft channels is several times larger than that in rigid nanochannels for certain parameters of grafting polymers. Jian *et al.*^16^ reported that, with the combined effects of wall softness and the viscoelastic rheology of fluids, grafting PELs to the channel surfaces improves the efficiency of EKECs under certain optimized parameters. Recently, Das and co-workers^38,39^ have reported that grafting end-charged PELs to the channel surfaces greatly improves the strength of the EOF. Different from previous work on the enhancement of flow by increasing the charge density of surfaces,^35^ the enhancement of flow originates from the spatial shift of ions in the EDLs by the end-charged PELs. In a series of works, Das and co-workers^40,41^ have shown that nanochannels grafted with end-charged PELs or poly-zwitterionic layers with certain properties (*e.g.* grafting density and length of PE) significantly improve the energy conversion efficiency of an EKEC, compared with brush-free nanochannels.

A reliable prediction of electrokinetic transport is required to estimate the optimal operation parameters of EKEC devices, which is crucial for the design of EKEC devices. Although the electrokinetic transport theory has been extensively studied,^42,43^ obscure points for electrokinetic transport over bare surfaces, such as flow reversal and the role of the Stern layer, still need to be clarified.^44^ Also, it is challenging to accurately predict the transport of fluids through channels grafted with polymer layers. The classic method is mostly based on the Navier–Stokes (NS) equation for flow, the Poisson–Boltzmann (PB) equation for the distribution of ions, and the Darcy equation for the drag experienced by flow through PELs. However, the method does not accurately model electrokinetic transport through PELs. Firstly, the conformation and hydrodynamic properties of PELs require advanced models, such as the molecular dynamic (MD) simulations and the lattice Boltzmann simulation.^45^ Netz *et al.*^46^ investigated the effect of electric field on the condensation of PEs and reported the scaling of critical field on the nonequilibrium unfolding of PEs. Secondly, the charged PE beads also affect the distribution of ions in the EDLs. Advanced theories, such as classical density functional theory (cDFT),^47^ are required to properly describe the delicate interplay between charged PE beads and ions. To understand the transport of fluids in PELs, MD simulations have been performed to obtain detailed descriptions of flow transport, distribution of ions, and conformation of polymers at molecular scales. Several groups have applied MD simulations to understand the EOF through polymers. Hickey *et al.*^48^ studied the EOF through charged polymers and observed that the direction of the EOF reverses well before the net charge of the interface (the wall and PELs) changes sign. Cao *et al.*^49^ studied the EOF through nanochannels with polymer patterning surfaces by MD simulations and observed that the polymer patterning induced anisotropy of the EOF when the direction of the electric field was changed. In a recent work, Das and co-workers^50^ studied the EOF through nanochannels functionalized with PE brushes by all-atom MD simulations and observed that the direction of the EOF changed by changing the electric field strength.

The above works shed light on the electrokinetic transport of flow in channels grafted with PELs. However, the mechanism for controlling the strength of the EOF is not clear and several key questions remain to be answered. Firstly, in the classic method, the hydrodynamic drag of PELs is accounted for by the friction coefficient governed by Darcy’s equation, and PELs are modeled as resistance centers to flows.^38^ However, PEs are a string of beads rather than isolated beads and their shielding effect strongly affects their hydrodynamic properties.^45,51^ How does a realistic model of PEs affect the variation of the EOF by end-charged PELs? Secondly, as the grafting density of the PEs varies, the conformation of the PEs varies from mushroom to brush-like. How does the conformation of the PEs affect the variation of the EOF by end-charged PELs? Resolving these problems is important to accurately predict the performance of EKEC devices with end-charged PELs. Herein, we study the flow transport through end-charged PELs by MD simulations. We observe that *V*_0_ follows a non-monotonic variation as the separation between PEs (*d*) decreases. As *d* decreases from 3.5 nm to 0.4 nm, the conformation of PEs changes from mushroom, to semi-dilute brush, and to concentrated brush. Furthermore, the variation of *V*_0_ strongly correlates with the conformation of the PEs. Specifically, as *d* decreases, *V*_0_ decreases in the mushroom conformation and then *V*_0_ increases in the concentrated brush conformation.

The rest of the manuscript is organized as follows. The methodology is introduced in Section 2. We propose a technique of velocity decomposition to quantify the competition between the driving effect from ions and the drag effect from PELs. In Section 3, we study flow transport through PELs with various separations and elucidate the mechanism and the structural origin of the variation of flow strength for PELs. We conclude in Section 4.

## 2 Methods

The EOF in a nanochannel grafted with PELs was simulated by MD simulations. The velocity profiles of the EOF, as well as density profiles of the solvent, ions, and PE beads across the nanochannels, were obtained from the MD simulations. The governing factors on the flow strength were analyzed by the Navier–Stokes–Brinkman (NSB) equation. The NSB equation was applied to compare the competition between the driving effect from net concentration of ions (*c*_ni_) and the drag effect from the PEs.

### 2.1 MD simulations

**Fig. 1:**
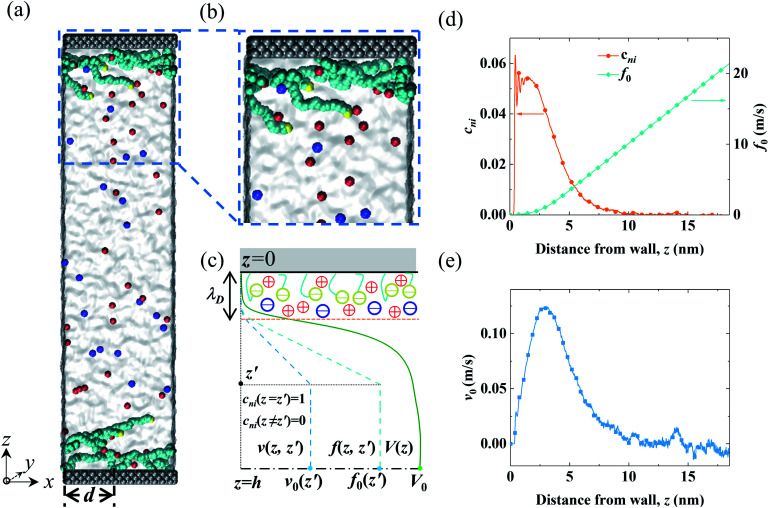
Snapshots from MD simulations and a sketch of the EOF velocity decomposition method. (a and b) A homogeneous electric field *E*_*x*_ was applied in the *x* direction to drive the EOF through the end-charged PELs grafted to the channel walls. The cations and anions are depicted by red and blue colors, respectively. The solvent is depicted as grey media. The charged wall atoms and end-charged PELs are depicted by the yellow color. The PELs with *N* = 24 mer were grafted with *d* = 3.5 nm. (c) A sketch of the decomposition of the EOF velocity *V*(*z*) into its components *v*(*z*,*z*′) by the velocity function *f*(*z*,*z*′) for a normalized pulse of *c*_ni_ at *z*′ (*i.e.*, *c*_ni_(*z* = *z*′) = 1 and *c*_ni_(*z* ≠ *z*′) = 0). The procedure for the decomposition of the EOF velocity is given in the main text. (d) The net concentration of ions *c*_ni_ and the velocity function of the flow strength *f*_0_ for PELs with *d* = 3.5 nm. (e) The components of the flow strength *v*_0_ generated by *c*_ni_. The snapshots of the MD systems were generated by the VMD package.^69^

The MD system consisted of an electrolyte confined between two parallel walls grafted with two types of polymers (end-charged PELs and neutral polymers). The snapshots of the MD systems are shown in Fig. 1(a) and (b). The ionic strength of the electrolyte (*I*_bulk_) was 3.4 × 10^−2^ mol L^−1^, corresponding to that in experiments (0.56 M).^52^ As the lateral sizes of the systems varied for polymers with various *d*, the number of ions in the system was different. *I*_bulk_ for systems with various *d* was set to be within a 5% difference. *d* varied from 0.4 nm to 3.5 nm. For *d* = 2.0 nm, the grafting density of the polymers (*σ*) was 0.164 nm^−2^, in agreement with that used in experiments.^52^ The walls were constructed by three layers of atoms placed on the FCC lattice with an atom density of 33.3 nm^−3^. For the neutral polymer system, the wall atoms in contact with the electrolyte were charged, with a surface charge density (*σ*_s_) of 3.28 × 10^−2^ C m^−2^, as used in experiments,^52^ and the atoms of the other layers were left uncharged. For the end-charged PE system, the non-grafted end-beads of PELs uniformly carried charges. The number of charges carried by the end-charged PE beads was the same as that in the neutral polymer system. The charge density of the PELs (*σ*_s,pel_) was equivalent to the surface charge density (*σ*_s_). Due to similar constraints from the sizes of the systems, *σ*_s,pel_ for systems with various *d* was controlled to be within a 5% difference.

Depending on the separation between polymers, the lateral dimensions (*x* and *y* directions) of the system varied from 5 nm to 7 nm. The channel widths (*w*) were 27 nm or 37 nm for systems with *d* > 1.5 nm or *d* < 1.5 nm, respectively. Such channel widths were wide enough for non-overlapping EDLs in the center of the channel. The strength of the EOF (*V*_0_) was measured from the EOF velocity at the center of the channel. *V*_0_ was independent of *w* for channels with non-overlapping EDLs.^53^*w* was the distance between the innermost layers of the two walls and *z* = 0 was chosen at the center of the innermost layer of the bottom wall. A vacuum space with a width of two times the channel width *w* (∼80 nm) was added in the *z* direction. The system parameters are summarized in Table S1.†

To focus on the hydrodynamic properties, we used the Weeks–Chandler–Andersen (WCA) potential^54^ for interactions among the solvent, PE beads, and the ions. We chose such a primitive model instead of an atomistic solvent model (*e.g.* SPC/E model) for the following reasons. First, it is well known that such a primitive solvent model can correctly capture the essential features of the EOF^54^ (*e.g.* ion distribution across the channel and flow strength of the EOF). Secondly, such a model neglects the chemical details of the solvent molecules and allows us to focus on the hydrodynamic interactions between the solvents and PE brushes. Finally, because of the high thermal noise in the EOF simulations by MD, a large external electric field (above 0.1 V nm^−1^ (ref. 54–57)) is applied to enhance the statistical accuracy. Such a strong electric field may result in the orientation of solvent molecules and may affect the interfacial properties of fluids, such as the viscosity. We have, therefore, chosen to use such a primitive model by modeling the solvent as non-polar WCA spheres, assuming a background dielectric constant, in order to avoid these complications. The dielectric constant was taken as *ε*_s_ = 78 to account for the dielectric properties of the solvent. We note that the permittivity of the solvent in a soft interface may be reduced due to confinement and collective polarization effects.^58,59^ However, only the out-of-plane dielectric constant of solvent is reduced, while the in-plane dielectric constant does not change much. We verified by separate simulations that the reduced dielectric constant does not strongly affect the net distribution of ions across the channel. Hence, for simplicity, here we took a fixed dielectric constant across the channel. The force field parameters for the solvent and ions were the same as those in ref. 60. Polymers were modeled by the united-atom model regarding the polyethylene glycol (PEG) molecule. The weighted atoms (carbon and oxygen) in each monomer (mer) of PEG (–CH_2_CH_2_O–) were represented by WCA spheres. The topology files for the polymers were obtained from the PRODRG2 server.^61^ Force field parameters for polymers were taken from the OPLS parameters for hydrocarbons.^62^ Dihedral parameters were tuned to obtain a flexibly deformed polymer. The detailed force field parameters were described in ref. 60.

MD simulations were performed using the simulation package GROMACS 4.5.1^62^. The cut-off radius of the Lennard-Jones (LJ) potential was 2^1/6^*σ* (*σ* = 0.3 nm) to model the solution conditions of polymers in a good solvent.^64^ Electrostatic interactions were computed using the particle mesh Ewald (PME) method in 2D (in *x* and *y* dimensions)^63^ with a real-space cut-off radius of 1.3 nm. To generate an EOF, an electric field was applied in the *x* direction with a strength of 0.08 V nm^−1^. This field strength was used to enhance the statistical accuracy and lies within the typical range of external electric fields (above 0.1 V nm^−1^) used in other MD simulations.^49,54–57^ In our previous work,^51^ we verified that the flow strength of the EOF increases linearly with the external electric field in a range up to 0.16 V nm^−1^. Therefore, the flow strength in this work holds the linearity with the strength of the external electric field. An NPT simulation of a box of electrolyte with a pressure of one bar was performed to obtain the equilibrium density of the electrolyte in bulk. In the planar system, the density of the electrolyte at the center of the channel is tuned to be the same with that in bulk to control the pressure of the system. The initial configurations of the system were built utilizing Packmol.^65^ The simulations of the planar system were performed in the NVT ensemble for 10 ns to reach a steady state, which were followed by a production run of 100 ns. The time step was 4 fs. The temperature was kept at 300 K by the V-rescaling thermostat.^63^ Some simulations were also performed with the Nose–Hoover thermostat applied in the orthogonal degrees of the flow direction and quite similar velocity profiles were obtained. In each case, we performed three independent simulation runs with different random seeding. Then, we averaged the results from the independent runs to reduce the fluctuation and obtained error bars for the reported data.

### 2.2 Navier–Stokes–Brinkman model

The NSB model was adapted from the model in ref. 66.1
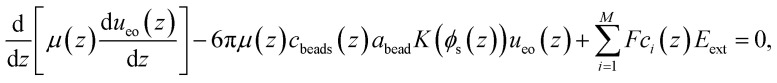
where *μ*(*z*) is the fluid viscosity, *u*_eo_ is the EOF velocity, *c*_beads_(*z*) is the number density of the PE beads, *a*_bead_ is the effective Stokes radius of the polymer beads, and *ϕ*_s_(*z*) is the volume of the polymer beads. The function *K*(*ϕ*_s_(*z*)) accounts for the correlations between homogeneously distributed spherical particles,^67^*F* is the Faraday constant, *c*_*i*_(*z*) is the ionic concentration of species *i*, *M* is the number of ionic species (*M* = 2), and *E*_ext_ is the applied electric field. On the left-hand side of eqn (1), the first term denotes the viscous force, the second term is the hydrodynamic drag exerted by the polymers on the fluid, and the last term is the driving force due to the external electric field. The driving force of the EOF is the net electrostatic force experienced by the ions (cations and anions) and its magnitude is in proportion to the net concentration of ions *c*_ni_ (*c*_ni_ = *c*_cat_ − *c*_ani_).

The concentrations of ions and PE beads (*c*_cat_(*z*), *c*_ani_(*z*), and *c*_beads_(*z*)) were measured directly in the MD simulations and used as inputs in eqn (1). The fluid viscosity (*μ*(*z*)) was obtained from a separate MD simulation using the method described in ref. 60. The model proposed by Batchelor and Green^68^ was used to take into account the variation of the viscosity with the position *z* across the channel. The no-slip boundary condition was applied at the wall and the mirror symmetry of the EOF velocity profile was imposed at the center of the channel. The hydrodynamic radius of the polymer beads (*a*_bead_), was treated as an adjustable parameter to match the EOF velocity profiles from the continuum model to those obtained by MD simulations.

### 2.3 EOF velocity decomposition

In light of Green’s function of Poisson’s equation, a velocity function technique was proposed to decompose the EOF velocity into its components generated by *c*_ni_. Due to the linearity of the NSB model (eqn (1)), the velocity profile (*V*(*z*)) generated by *c*_ni_ across the channel could be decomposed into the components of velocity (*v*(*z*,*z*′)) generated by *c*_ni_ at *z*′. The integral of *v*(*z*,*z*′) over the channel (*z*′ ∈ (0, *H*/2)) results in *V*(*z*). A schematic sketch of the velocity function technique is shown in Fig. 1(c). The velocity function (*f*(*z*,*z*′)) was the velocity generated by a normalized pulse of *c*_ni_ at *z*′ (*i.e.*, *c*_ni_(*z*′) = 1 and *c*_ni_(*z* ≠ *z*′) = 0). Due to the linearity of the NSB model, *v*(*z*,*z*′) was obtained by scaling *f*(*z*,*z*′) with *c*_ni_(*z*′), *i.e.*, *v*(*z*,*z*′) = *c*_ni_(*z*′)*f*(*z*,*z*′).

As for *V*_0_, its velocity function (*f*_0_(*z*′)) was computed to obtain its components (*v*_0_(*z*′)). Physically, *f*_0_(*z*′) quantified the capacity of *v*_0_(*z*′) generated by a normalized pulse of *c*_ni_ at *z*′. Fig. 1(d) showed that *f*_0_(*z*′) linearly increased with the position of ions *z*′ beyond a threshold location, which confirmed the dependency of the capacity of the generated *v*_0_(*z*′) on the ions position. With calculated *f*_0_(*z*′), *v*_0_(*z*′) can be obtained by scaling *f*_0_(*z*′) with *c*_ni_(*z*′) (Fig. 1(e)). *V*_0_ is integrated by *v*_0_(*z*′) (*z*′ ∈ [0, *H*/2]). The mathematical formulas of the flow strength decomposition are as follows.2
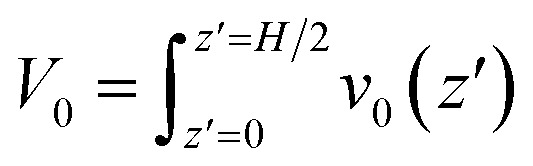

3 *v*_0_(*z*′) = *f*_0_(*z*′)*c*_ni_(*z*′)

For the same *c*_ni_, we get the same value of *V*_0_*via* integration of *v*_0_(*z*′) and direct solution from the NSB model (shown in Fig. S2(a)†). Besides, the same EOF velocity profile can be derived by these two methods. A detailed derivation of the EOF velocity decomposition is described in Section S2 of the ESI.†*f*(*z*,*z*′) at various *z*′ are shown in Fig. S2(b)† and *V* assembled by *f*(*z*,*z*′) is shown in Fig. S2(c).†

## 3 Results and discussion

Our primary interest is to clarify the mechanism governing the strength of the EOF in the channels grafted with end-charged PELs. As shown in Fig. 1(a) and (b), the distribution of end-charged PE beads results in the spatial shift of ions. The position of end-charged PE beads is related to the conformation of PEs, which is determined by the relative magnitude between the size of the PEs (*e.g.* the gyration radius, *R*_g_) and *d*. In the following subsections, we examine the EOF in channels grafted with end-charged PELs with various *d* to elucidate the factors governing the strength of the EOF.

### 3.1 The effect of grafting density on the flow strength

**Fig. 2:**
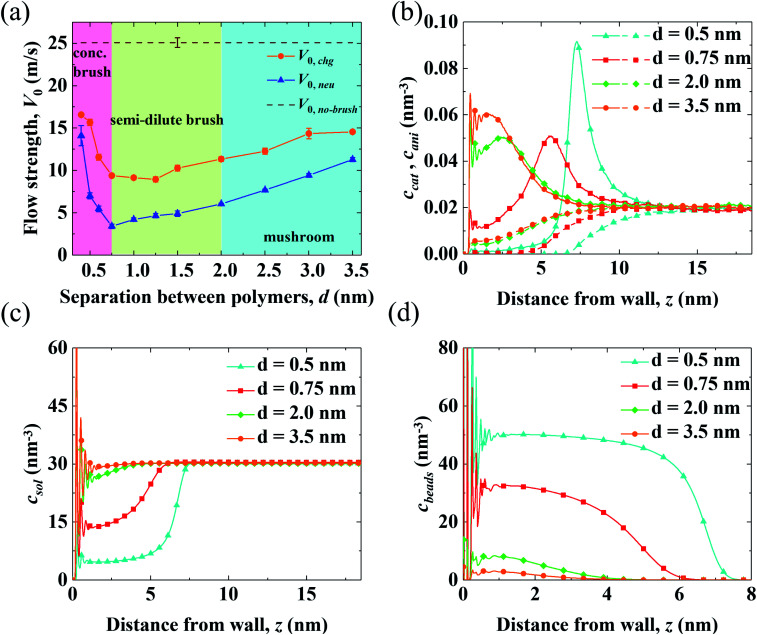
The EOFs through neutral polymers and end-charged PELs in nanochannels with *d* = 0.5, 0.75, 2.0, and 3.5 nm from MD simulations. (a) The variation of flow strength through end-charged PELs (*V*_0,chg_) and neutral polymers (*V*_0,neu_) with various *d*. The blue, green, and pink shadow areas in the plot denote the PEs at mushroom, semi-dilute brushes, and concentrated brushes regimes, respectively. *V*_0_ through the channel with no brushes is shown as a dash line. (b) The concentration of cation (*c*_cat_) and anion (*c*_ani_) across the channel in end-charged PE systems, with the cation shown in solid line and anion shown in dash line. (c and d) The concentration of solvent *c*_sol_ (c) and PE beads *c*_beads_ (d) across the channel in end-charged PE systems.

Results for the EOF in a channel grafted with end-charged PELs with various *d* (*d* = 0.4–3.5 nm) are shown in Fig. 2. As *d* decreases from 3.5 to 0.4 nm, the conformation of the PEs changes from mushroom, to semi-dilute brush, and to concentrated brush (characterized by a criteria from neutral polymer brushes,^21^ see Section S1 of the ESI† for details). Concentrated PE brushes refer to the self-assembled monolayer (SAM) layers in experiments.^70^

The variations of flow strength through end-charged PELs (*V*_0,chg_) and neutral polymers (*V*_0,neu_) are shown in Fig. 2(a). We observe that both *V*_0,chg_ and *V*_0,neu_ follow a non-monotonic variation. In the mushroom regime (2.0 < *d* < 3.5 nm), both *V*_0,chg_ and *V*_0,neu_ decrease as *d* decreases. In the semi-dilute brush regime (0.75 < *d* < 2.0 nm), *V*_0,chg_ remains almost constant while *V*_0,neu_ decreases as *d* decreases. In the concentrated brush regime (0.4 < *d* < 0.75 nm), both *V*_0,chg_ and *V*_0,neu_ increase. The variation of *V*_0_ as a function of *d* originates from the competition between the spatial shift of *c*_ni_ in EDLs and the drag from PELs and will be discussed in the subsequent section. Generally, *V*_0,chg_ is larger than *V*_0,neu_ over the whole range of *d*. As shown in Fig. S3,† the ions are partly attracted to the charged walls in the neutral polymer systems, while the ions are attracted to the charged PE beads in the end-charged PE system. Hence, the driving forces in the neutral polymer system partially decreases, resulting in a weaker *V*_0_. In addition, the increase of *V*_0,neu_ is larger than that of *V*_0,chg_ for polymers with the concentrated brush conformation and *V*_0,neu_ approaches *V*_0,chg_ at *d* = 0.4 nm. As shown in Fig. S3(a)–(c),† the peak of the counter-ions reduces for neutral polymers with the concentrated brush conformation, due to the steric effect from the polymers. For polymers with *d* = 0.4 nm, the distribution of ions between the neutral polymer system and end-charged polymer system is comparable because ions are expelled from the polymers at such grafting density.

To clarify the variation of *V*_0,chg_, we examine the concentration of cations (*c*_cat_) and anions (*c*_ani_) across the channel in end-charged PE systems with various *d*, as shown in Fig. 2(b). Several separations of PELs (*d* = 0.5, 0.75, 2.0, and 3.5 nm) are chosen to represent the PEs in the mushroom, semi-dilute brush, and concentrated brush regimes. Compared with those in the neutral polymer system (Fig. S3†), *c*_cat_ and *c*_ani_ in the end-charged PE system are distributed in a wider region due to the attraction from the end-charged PE beads. As *d* decreases, cations move away from the walls with an enhanced peak. For PELs with the concentrated brush conformation (*d* < 0.75 nm), cations form a single peak at the interface between the PELs and the fluid. The distributions of *c*_cat_ and *c*_ani_ originate from a combination of the electrostatic interaction from the charged PE beads and the steric effect from the PELs. Ions are attracted to the charged PE beads due to electrostatic attraction. Additionally, ions move further away from the wall due to the steric interaction with the PELs.

The distributions of solvents (*c*_sol_) and PE beads (*c*_beads_) across the channel in end-charged PE systems with various *d* are shown in Fig. 2(c) and (d). *c*_sol_ and *c*_beads_ are corrolated with the conformation of the PELs. For PELs in the mushroom regime (*d* > 2.0 nm), *c*_sol_ is barely affected by *c*_beads_. For PELs in the concentrated brush regime (*d* < 0.75 nm), as *c*_sol_ in region of PELs (0–5 nm) reduces from ∼30 nm^−3^ to ∼15 nm^−3^, *c*_beads_ in the same region of PELs increases from ∼10 nm^−3^ to ∼30 nm^−3^. As *d* further decreases to 0.4 nm, *c*_sol_ in the region of PELs reduces to ∼6 nm^−3^ and *c*_beads_ in the region of PELs increases to ∼50 nm^−3^. The repulsion of solvents from the PELs is ascribed to the steric effect from the PE beads for the PELs with concentrated brush conformation.

### 3.2 The mechanism for the variation of flow strength

**Fig. 3:**
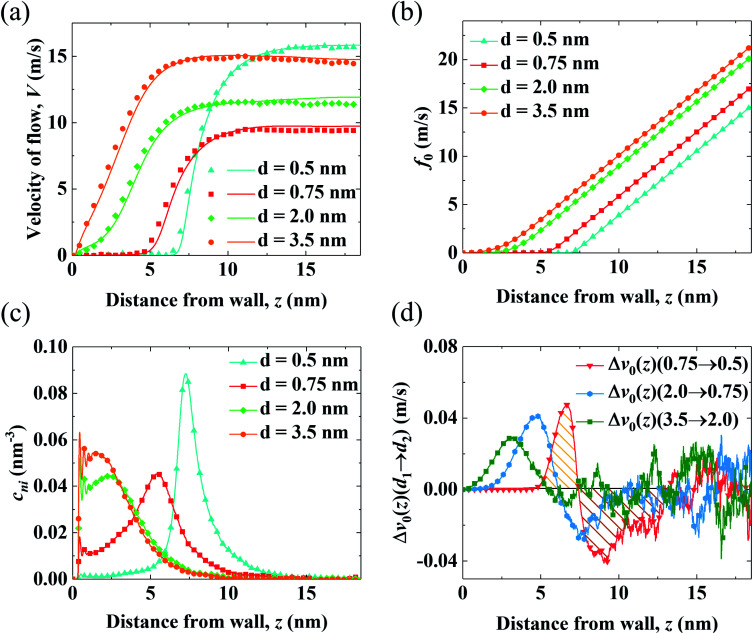
Competition between the shift of *c*_ni_ and *f*_0_ in end-charged PE systems with various *d*. (a) Velocity profiles from MD simulations (shown by markers) are compared with those predicted by continuum NSB model (shown by solid lines with the same colors). (b) Velocity function of the EOFs through PELs with various separations *d* (*f*_0,*d*_(*z*)). (c) Distribution of *c*_ni_. (d) The change of the components of flow strength (*v*_0_) from *d*_1_ to *d*_2_ (Δ*v*_0_(*z*)(*d*_1_ → *d*_2_)).

To determine the mechanism for the variation of flow strength in end-charged PE systems with different *d*, the continuum NSB model is applied to quantify the driving and drag effect on the variation of *V*_0_ (Fig. 3). Fig. 3(a) shows the velocity profiles obtained from the MD simulations and those predicted by the NSB model, which shows that the predicted velocity profiles from the NSB model are in good agreement with those from the MD simulations even in the concentrated brush regime, hence the NSB model is used to elucidate the factors governing the flow strength. Other velocity profiles for different *d* are shown in Fig. S4.†

We apply the method of velocity decomposition to quantify the competition between the spatial shift of *c*_ni_ and drag from PELs. The procedure of velocity decomposition is described in the Methods section. Essentially, *V*_0_ is decomposed into its components (*v*_0_) perturbed by *c*_ni_ at *z*′. *v*_0_(*z*) can be quantified through the velocity function (*f*_0_) by scaling *f*_0_(*z*) with *c*_ni_(*z*), *i.e.*, *v*_0_(*z*) = *c*_ni_(*z*)*f*_0_(*z*) and the distribution of *c*_ni_ for various *d* (*d* = 0.5–3.5 nm) are shown in Fig. 3(b) and (c), respectively. For *f*_0_ at the same separation, *f*_0_(*z*) linearly increases as position *z* moves away from the walls, which shows that the spatial shift of *c*_ni_ away from the walls results in a stronger driving effect. For the velocity function with different *d* (*f*_0,*d*_), *f*_0,*d*_ decreases as *d* decreases due to the increased drag. For the distribution of *c*_ni_ in end-charged PE systems with various *d* (Fig. 3(c)), they show distinct features depending on the conformation of the PELs. Specifically, *c*_ni_ remains almost constant for PELs in the mushroom regime (*d* = 2.0–3.5 nm) and it shifts away from the walls by a larger magnitude for PELs in the brush-like regime (*d* = 0.5–2.0 nm).

To elucidate the competition between the spatial shift of *c*_ni_ and drag from the PELs, the change of *v*_0_ (Δ*v*_0_(*d*_1_ → *d*_2_)) as *d* varies is plotted in Fig. 3(d). Specifically, Δ*v*_0_(*z*)(*d*_1_ → *d*_2_) denotes the change of *v*_0_(*z*) as *d* decreases from *d*_1_ to *d*_2_, *i.e.*, Δ*v*_0_(*d*_1_ → *d*_2_) = *v*_0_(*d*_1_) − *v*_0_(*d*_2_). The positive part of Δ*v*_0_(*z*)(*d*_1_ → *d*_2_) results in a decrease of *V*_0_ (*V*_0,*d*1_ > *V*_0,*d*2_) and the negative part of Δ*v*_0_(*z*)(*d*_1_ → *d*_2_) results in an increase of *V*_0_ (*V*_0,*d*1_ < *V*_0,*d*2_). The change of *v*_0_(*z*) shows unique features depending on the conformation of the PELs. In the mushroom regime, the positive part of Δ*v*_0_(*z*)(3.5 → 2.0) spans from 2 nm to 3 nm and its negative part is almost zero, which shows that the drag from the PELs increases by a larger magnitude than the spatial shift of *c*_ni_, as supported by a constant position of *c*_ni_ for *d* = 3.5–2.0 nm. In the semi-dilute brush regime, the positive part of Δ*v*_0_(2.0 → 0.75) is comparable to its negative part, which implies that the increase of drag is comparable to the spatial shift of *c*_ni_, in line with a moderate shift of *c*_ni_ for *d* = 2.0–0.75 nm. In the concentrated brush regime, the positive part of Δ*v*_0_(0.75 → 0.5) is smaller than the negative part, which indicates that the drag from the PELs increases by a smaller magnitude than the spatial shift of *c*_ni_, as supported by a significant shift of *c*_ni_ with an enhanced peak for *d* = 0.75–0.5 nm.

### 3.3 Structural analysis of PELs with various separation

**Fig. 4:**
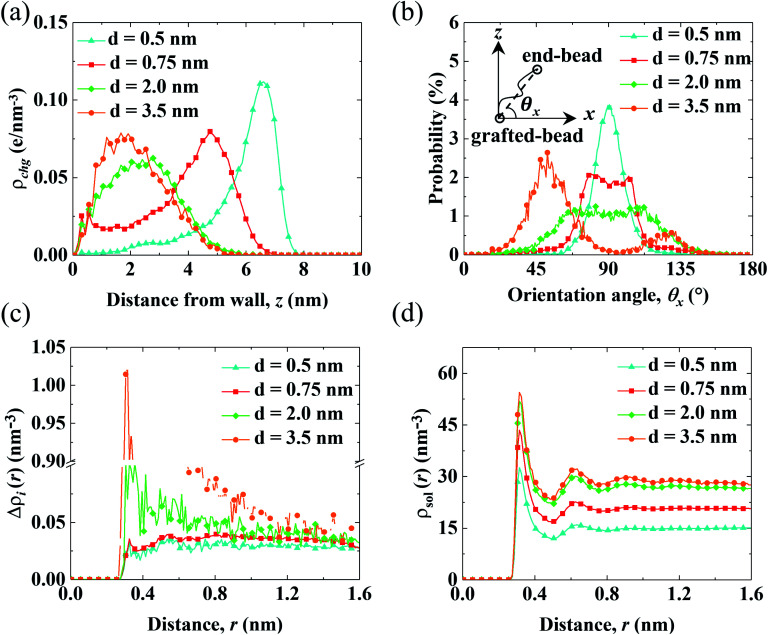
Structural properties of the PELs with various *d*. (a) Distribution of the charges of the PELs (*ρ*_chg_). (b) Probability distribution of the parallel orientation angle (P(*θ*_*x*_)). (c and d) Net radial number density of ions (Δ*ρ*_i_(*r*)) (c) and radial number density of solvents (*ρ*_sol_(*r*)) (d) around end-charged PE beads.

The aforementioned analysis shows that the spatial shift of *c*_ni_ correlates with the variation of *V*_0_. Meanwhile, *c*_ni_ is affected by the conformation of the PELs due to the electrostatic attraction from the end-charged PE beads. Therefore, we analyze the structure of the PELs to elaborate upon the structural origin of *c*_ni_ in Fig. 4.

Fig. 4(a) shows the distribution of the charges of the PELs (*ρ*_chg_) in systems with various *d*. The distribution of *ρ*_chg_ is calculated by the volume charge density of the end-charged PELs. The distribution of *ρ*_chg_ shows similar features to *c*_ni_. Therefore, the distribution of *ρ*_chg_ is analyzed to elaborate the structural origin of *c*_ni_. As *d* decreases, the spatial shift of *ρ*_chg_ increases as the space available for the polymer beads to pack decreases. Specifically, *ρ*_chg_ shifts by a small amount in the mushroom conformation since there is enough space for the PE beads to pack. The spatial shift of *ρ*_chg_ increases in the brush-like conformation. *ρ*_chg_ shift by a significant amount in the concentrated brushes conformation. This can be ascribed to the dense layer formed by the polymers where ions and solvents get expelled from PELs. Under such confinement the PE beads have insufficient space to pack in the polymers layer.

To elucidate the arrangement of PEs, the distribution of the parallel orientation angle (*θ*_*x*_) is calculated in Fig. 4(b), where *θ*_*x*_ is the angle formed between the orientation vector of the PEs and the unit vector of the *x* axis. The orientation vector of the PEs points from the grafted bead at the wall to the end-charged bead. As *d* decreases, the probability of *θ*_*x*_ (*P*(*θ*_*x*_)) shifts from 45° to 90°, corresponding to the unfolding of PEs from the mushroom to the brush-like conformation. For polymers with *d* = 3.5 nm, *P*(*θ*_*x*_) at 45° is higher than that at 135°, due to the inducement of flow along the *x* axis. The probability of the perpendicular orientation angle (*θ*_z_) (*P*(*θ*_*z*_)) changes from 45° to 0° (shown in Fig. S5(a)†), corresponding to the unfolding of the PEs from the mushroom to brush-like conformation.

The solvation of end-charged beads by species is quantified by the net radial number density of ions(Δ*ρ*_i_(*r*)), the radial number density of PE beads (*ρ*_beads_(*r*)) and solvents (*ρ*_sol_(*r*)) around the end-charged PE beads, as shown in Fig. 4(c) and (d) and S5b.† Δ*ρ*_i_(*r*) and *ρ*_sol_(*r*) show different features which are strongly dependent on the conformation of the PEs. In the mushroom regime, as *d* decreases, Δ*ρ*_i_(*r*) reduces by a large amount (the value of first peak of Δ*ρ*_i_(*r*) reduces by a factor of ∼10), while *ρ*_sol_(*r*) slightly reduces. This is beacuse that as *d* decreases, the amount of end-charged PE beads increases by a factor of ∼3, while the amount of *c*_ni_ remains constant, resulting in a significant reduction of Δ*ρ*_i_(*r*) in the mushroom conformation. A sufficient amount of solvents surrounds the end-charged PE beads in the mushroom conformation, resulting in a slight reduction of *ρ*_sol_(*r*) in such conformation. The variation of Δ*ρ*_i_(*r*) and *ρ*_sol_(*r*) in the mushroom regime shows that the space in the PELs in such regime is large enough that solvents and ions are not expelled from the PELs. In the concentrated brush conformation, as *d* decreases, Δ*ρ*_i_(*r*) decreases by a small amount, while *ρ*_sol_(*r*) is reduced by a large amount (the value of first peak of *ρ*_sol_(*r*) reduces by ∼50%). This is because that as *d* decreases, the amount of charged PE beads increases by a factor of ∼2, while the amount of ions around the end-charged PE beads also increases due to the enriched ions expelled from PELs, resulting in a weak decrease of Δ*ρ*_i_(*r*) in the concentrated brush conformation. Solvents are expelled from the PELs and the amount of solvents around the end-charged PE beads reduces, resulting in a large reduction of *ρ*_sol_(*r*) in the concentrated brush conformation. The variation of Δ*ρ*_i_(*r*) and *ρ*_sol_(*r*) in the concentrated brush conformation shows that the space in the PELs with such conformation is so limited that species are expelled from the PELs. The evolution of the solvation structure of the end-charged beads is consistent with the spatial shift of *c*_ni_ and they are all correlated with the conformation of PEs.

### 3.4 Other factors affecting the variation of flow strength

#### 3.4.1 Flow enhancement by end-charged PELs

In the MD systems, *V*_0_ in channels grafted with PELs is weaker than that with no brushes, which is different from other studies.^38,40,41^ This may be due to the short polymer length that can be modeled by the current MD simulations. The polymer lengths (*N* = 2000 mer) in other studies^38,40,41^ are much larger than that in the current system (*N* = 24 mer).

#### 3.4.2 The effect of PELs with fixed end-charged PE beads on the flow strength

In continuum models,^16,38^ the PELs are usually modeled as fixed polymer beads. While in MD simulations, PELs are modeled as freely moving polymers. *V*_0_ through PELs with fixed end-charged PE beads (fixed-PELs) and freely moving end-charged PE beads (freely-PELs) are shown in Fig. S6.† The end-charged PE beads in the fixed-PEL system are at the peak position of *ρ*_chg_ in the freely-PEL system. A comparison of *c*_beads_ between fixed-PEL system and freely-PEL system is shown in Fig. S7.† Fig. S6† shows that *V*_0_ through fixed-PELs is stronger than that through freely-PELs in the brush-like regime, which originates from a more concentrated distribution of *c*_ni_ at the fixed-PELs system, leading to a stronger driving effect. In the mushroom regime, *V*_0_ through fixed-PELs is comparable with that through freely-PELs. This behavior may be caused by the fact that ions are less affected by the position of charged PE beads because there is more space available in PELs with the mushroom conformation.

#### 3.4.3 The effect of the space charge density of PE brushes on the flow strength

In fact, the charged functional groups may not be at the tail of a PE brush. We studied the effect of charged polymer beads distributed at the middle and in the tail of the PE brushes. The results show that *V*_0_ through such PE brushes is generally weaker than that through the end-charged PE brushes (see Section S3 of the ESI† for details).

#### 3.4.4 The effect of the ionic strength of the solution on the flow strength

The ionic strength of the electrolyte solution is another factor affecting the variation of flow strength. We examine the effect of ionic strength on the flow strength, as shown in Fig. S9.† Due to the computational limit, the effect of ionic strength is examined by increasing the ionic strength *I* by a factor of 3.5, 5, and 7.5. The results show that *V*_0_ decreases as the ionic strength increases for PELs in the mushroom conformation. Such an observation agrees with previous work for cases with short polymer lengths.^38^ In the current system, as the ionic strength increases, *c*_ni_ shifts towards the walls, resulting in a weaker driving effect of *c*_ni_ and the decrease of *V*_0_.

#### 3.4.5 Hydrodynamic radius of the PE beads

The hydrodynamic radius of the PE beads (*a*_bead_) is the only fitting parameter used by the NSB model to match the velocity profiles of MD simulations. We examine the *a*_bead_ of end-charged PELs with various *d* in Fig. S10.†*a*_bead_ is around 0.1 of its physical size *a*_0_ (*i.e.*, LJ size and *a*_0_ = 0.156 nm) for PELs with the mushroom conformation, in agreement with our previous work.^51^ As *d* decreases, *a*_bead_ increases until ∼0.5*a*_0_ for PELs with the concentrated brush conformation. The increase of *a*_bead_ may be due to the unfolding of PEs from mushroom to brush-like configuration. From our previous work,^51^*a*_bead_ in the brush-like configuration is larger than that in mushroom configuration.

## 4 Conclusions

We have studied the variation of *V*_0_ in nanochannels grafted with end-charged PELs using MD simulations, analyzed by the continuum NSB model. We observe that *V*_0_ follows a non-monotonic variation as *d* decreases from 3.5 nm to 0.4 nm. *V*_0_ decreases first as 2.0 < *d* < 3.5 nm, in agreement with previous studies.^38^ In particular, *V*_0_ increases as *d* decreases for PELs with a concentrated brush conformation (*d* < 0.75 nm), a new behavior of flow strength.

The variation of *V*_0_ through PELs with *d* results from the competition between the increased drag from PELs and the spatial shift of *c*_ni_ away from the walls. A method using the velocity function (*f*_0_) is proposed to decompose *V*_0_ into *v*_0_ (the components of *V*_0_ perturbed by *c*_ni_). Δ*v*_0_ (the change of *v*_0_) is examined to quantify the competition between the driving effect from *c*_ni_ and the drag effect from PELs. For PELs in the mushroom regime (2.0 < *d* < 3.5 nm), the drag from PELs dominates over the driving effect from the spatial shift of *c*_ni_, resulting in the decrease of *V*_0_. For PELs in the concentrated brush regime (*d* < 0.75 nm), the driving effect from the spatial shift of *c*_ni_ dominates over the drag from PELs, resulting in the increase of *V*_0_. The structural analysis of the PELs shows that *ρ*_chg_ is highly correlated with the distribution of *c*_ni_. The distribution of *ρ*_chg_ is rationalized by the conformation of the PELs. In the mushroom regime, there is enough space in the PELs for the PE beads to pack, therefore *ρ*_chg_ changes little as *d* decreases. In the concentrated brush conformation, less space is available in the PELs for the PE beads to pack, resulting in the expulsion of ions and solvents from the PELs, therefore *ρ*_chg_ shifts by a large amount as *d* decreases. The radial density distribution of species also demonstrates that enough spaces are available in the PELs with the mushroom conformation for ions and solvents to pack, leading to reduced ions around the end-charged PE beads. While there is less space available in the PELs with the brush conformation, resulting in the expulsion of ions from the PELs and enhanced ions around the end-charged PE beads. Overall, the variation of *V*_0_ through end-charged PELs originates from the interplay between the structure of the PELs and the distribution of ions. Although the enhancement of the electrokinetic transport over concentrated brush-like PELs has not been validated by experimental measurements, it is well known that high packing densities are crucial for the tribological performance of monolayers.^71–73^ Our theoretical work suggests that densely packed monolayers can enhance electrokinetic transport and encourages further experimental studies.

## Author contributions

P. W.: conceptualization, funding acquisition, supervision and writing – original draft; T. S.: software, data curation, visualization, investigation and writing – review & editing; X. J.: investigation and writing – review & editing.

## Conflicts of interest

There are no conflicts to declare.

## Supplementary Material

RA-012-D1RA06601C-s001
