# Supplementary material for: Non-monotonic variation of flow strength in nanochannels grafted with end-charged polyelectrolyte layers
Source: RSC Adv. 2022 Feb 2;12(7):4061–71. doi: 10.1039/d1ra06601c (PMC8981053; doi:10.1039/d1ra06601c)
Supplement: RA-012-D1RA06601C-s001 [file RA-012-D1RA06601C-s001.pdf]

Electronic Supplementary Material (ESI) for RSC Advances.
This journal is © The Royal Society of Chemistry 2022

Supplementary Information

Non-monotonic Variation of Flow Strength in Nanochannels Grafted with End-charged Polyelectrolyte Layers

Peng Wu,[*,†,‡] Tao Sun,[‡] and Xikai Jiang[¶]

†*School of Energy and Power Engineering, Inner Mongolia University of Technology, Hohhot, Inner Mongolia, 010051, China*

‡*China-EU Institute of Clean and Renewable Energy, Huazhong University of Science and Technology, Wuhan, Hubei 430074, China*

¶*State Key Laboratory of Nonlinear Mechanics, Institute of Mechanics, Chinese Academy of Sciences, Beijing 100190, China*

E-mail: wupeng@imut.edu.cn

Conformation of polyelectrolytes with various separations

The conformation of polymers can be categorized into mushroom, semi-dilute brushes, and concentrated brushes configurations.[1] According to Ref.,[1] the height of polymers h follows a power law with grafting density of polymers σ_{poly} (number of chains per area) governing by eqn (S2). The height of polymers h is defined as first moment of their number density c_{beads} with their position $h(z)$ away from walls, governing by eqn (S1). The exponent factor λ depends with the conformation of polymers. For polymers with mushroom conformation, $\lambda \sim 0$; for polymers with semi-dilute brush conformation, $\lambda \sim 1/3$; for polymers with concentrated brush conformation, $\lambda > 1/3$. The scaling of $\ln h$ with $\ln \sigma_{poly}$ is shown in Fig. S1(a). The exponent factor λ between every grafting density is computed by eqn (S3), as shown in Fig. S1(b). For $3.5 > d > 2.0$ nm, $\lambda \sim 0.1$ corresponds to PELs with mushroom conformation; for $2.0 > d > 0.75$ nm, $0.3 > \lambda > 0.1$ corresponds to PELs with semi-dilute brush conformation; for $0.75 > d > 0.4$ nm, $0.5 > \lambda > 0.3$ corresponds to PELs with concentrated brush conformation.

$$h = \frac{\int_0^{H/2} h(z) c_{beads}(z)}{\int_0^{H/2} c_{beads}(z)} \tag{S1}$$

$$h \sim N\sigma_{poly}^{\lambda}, \sigma_{poly} = d^{-2} \tag{S2}$$

$$\lambda = \frac{\Delta \ln h}{\Delta \ln \sigma_{poly}} \tag{S3}$$

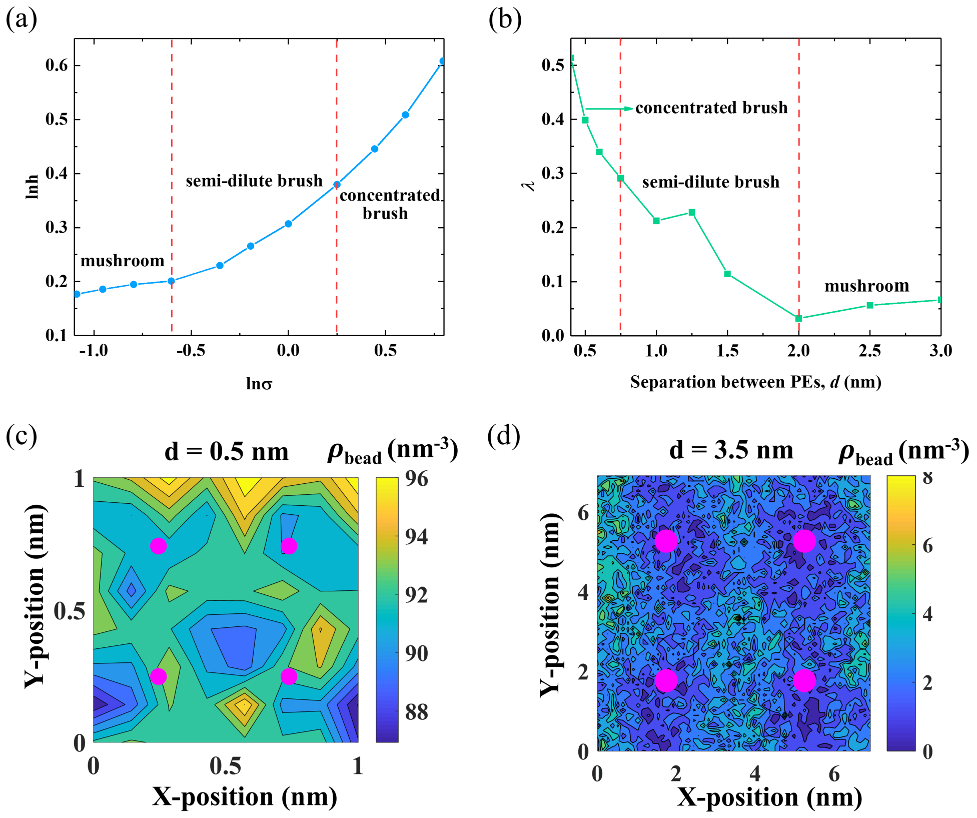

Figure S1: **Conformation of polyelectrolytes with various separations.** (a) The variation of the height of PEs $\ln h$ with their grafting density $\ln \sigma_{poly}$. (b) The variation of the exponent factor λ between every grafting density as a function of the separation d. (c-d) 2D density distribution of PE beads for $d = 0.5$ nm (c) and $d = 3.5$ nm (d). The grafting positions of PEs are shown in pink points. For $d = 3.5$ nm, the PELs show features of mushroom conformation while for $d = 0.5$ nm, the PELs are densely packed referring to concentrated brush conformation.

EOF velocity decomposition

Due to the linearity property of the NSB model, the EOF velocity across channel $V(z)$ can be decomposed into the components of EOF velocity $v(z, z')$ generated by c_{ni} at z' with $z' \in [0, H/2]$. Due to the linearity property of the NSB model, the EOF velocity generated by c_{ni} follows as:

$$v(z, z') = f(z, z')c_{ni}(z') \tag{S4}$$

$$V(z) = \int_{z'=0}^{z'=H/2} v(z, z') \tag{S5}$$

For $z' = H/2$, $v(z, z')$ is denoted as components of flow strength $v_0(z')$. The flow strength generated by c_{ni} follows as:

$$v_0(z') = f_0(z')c_{ni}(z') \tag{S6}$$

$$V_0 = \int_{z'=0}^{z'=H/2} v_0(z') \tag{S7}$$

The velocity profiles constituted by $v(z, z')$ and c_{ni} for $d = 0.75$ nm are shown in Fig. S2(c). The flow strength V_0 is calculated by integration of flow strength components $v_0(z)$ and they match well with the flow strengths directly solved by NSB model for the same c_{ni} for PELs system with $d = 0.75$ nm.

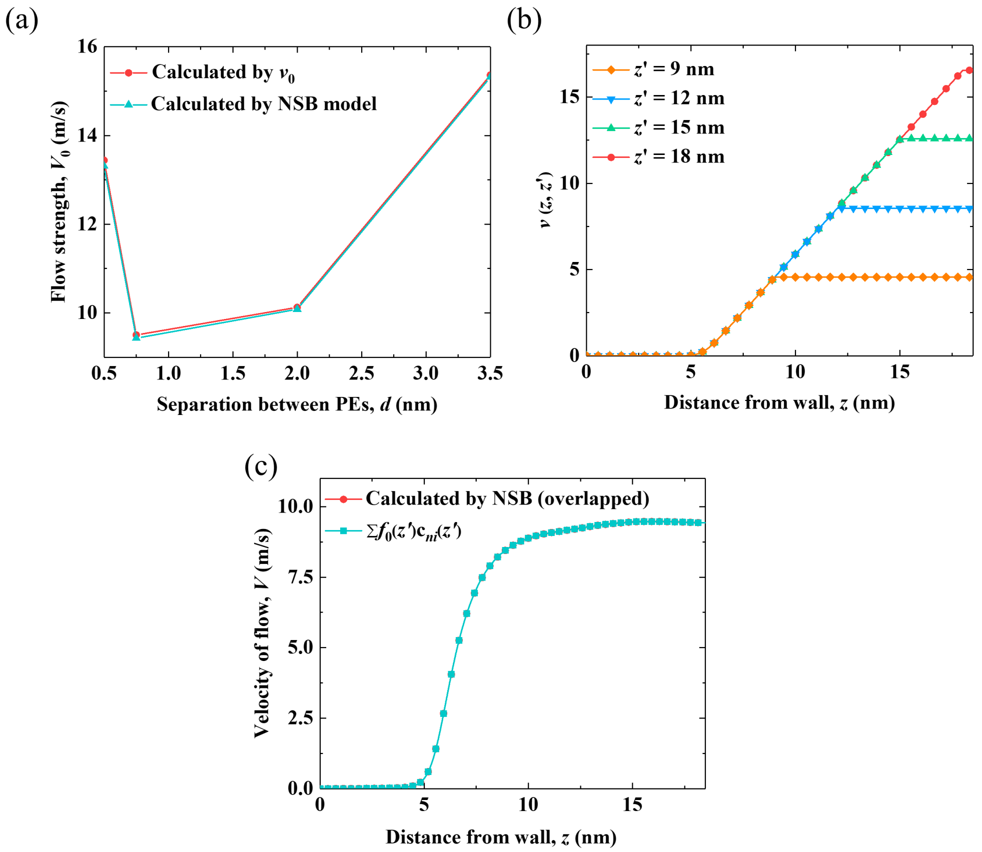

Figure S2: **Velocity profiles compared by flow velocity decomposition and NSB model.**(a) The comparison of flow strength V_0 solved by NSB model and that constituted by flow strength components $v_0(z)$. (b) The components of EOF velocity $v(z, z')$ by c_{ni} at different z'. (c) The comparison of the velocity profiles solved by NSB model and that constituted by the components of EOF velocity $v(z, z')$.

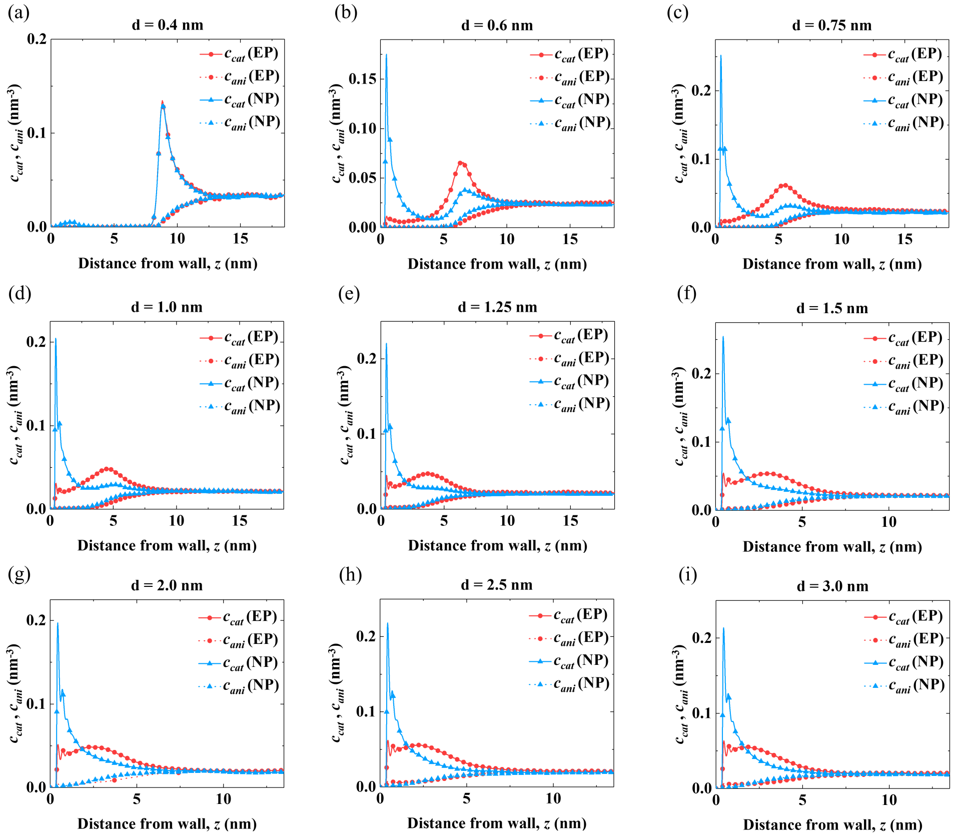

Figure S3: **Comparison of the concentrations of ions (cation c_{cat} and anion c_{ani}) in end-charged PE (EP) and neutral polymers (NP) systems with $d = 0.4 - 3.0$ nm.**

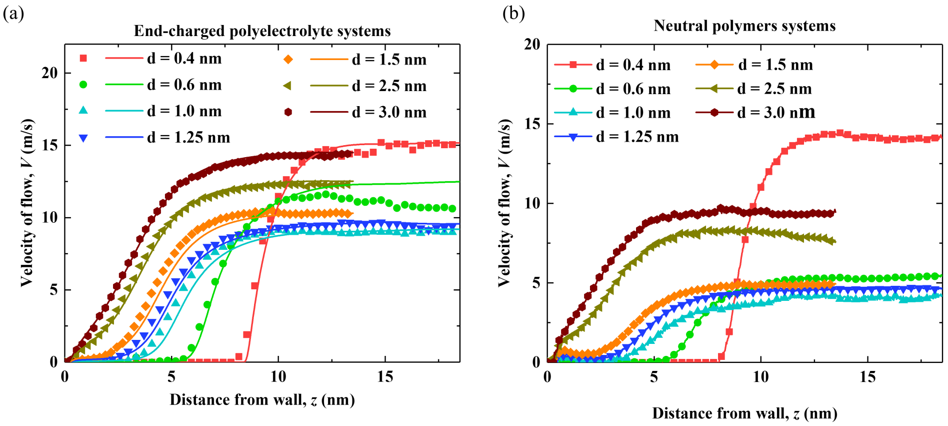

Figure S4: **Velocity profiles from MD simulations and the NSB model in end-charged PE (EP) and neutral polymers (NP) systems.** The velocity profiles in EP systems (a) and NP systems (b) with various d. Velocity profiles from MD simulations are shown in markers and that from the NSB model are shown in solid lines with the same colors. Due to the symmetry of velocity profiles, only half of the velocity profiles are plotted. To ensure non-overlapped EDLs in the channel, the channel width is set to be 27 nm for $d > 1.5$ nm and to be 37 nm for $d < 1.5$ nm.

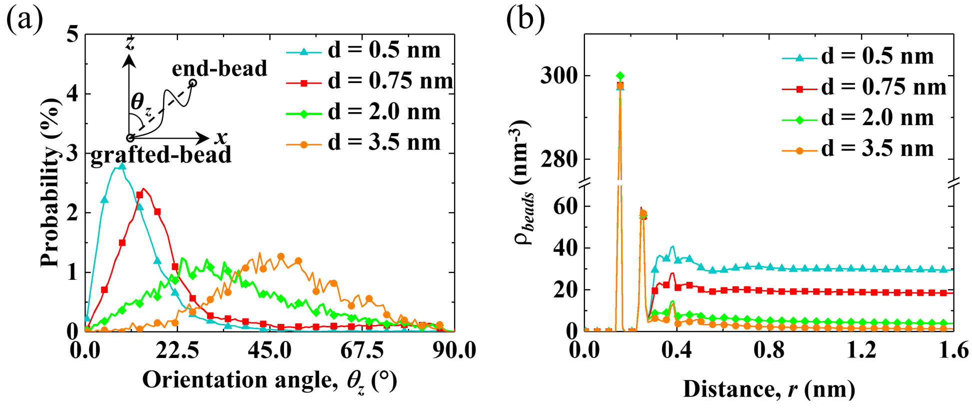

Figure S5: **Structural properties of PELs with various separation d.** (a) Probability distribution of perpendicular orientations angle (θ_z). θ_z is the angle formed between the orientation vector of PEs and the unit vector of z axis. (b) Radial number density of PE beads ρ_{beads} around the charged polymer beads.

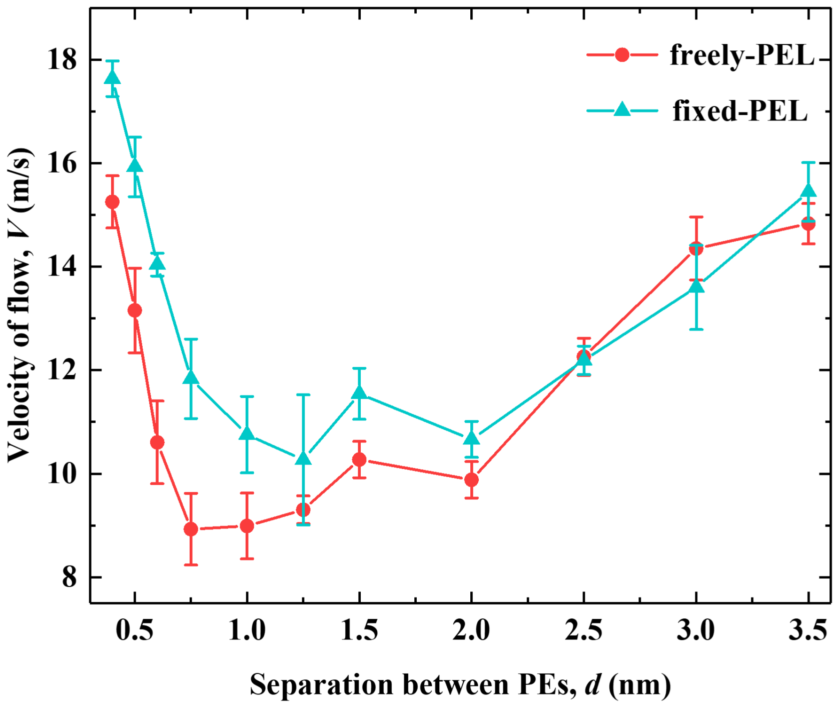

Figure S6: **Comparison of the flow strength V_0 through PELs with fixed end-charged polymer beads (fixed-PELs) and freely moved end-charged polymer beads (freely-PELs).**

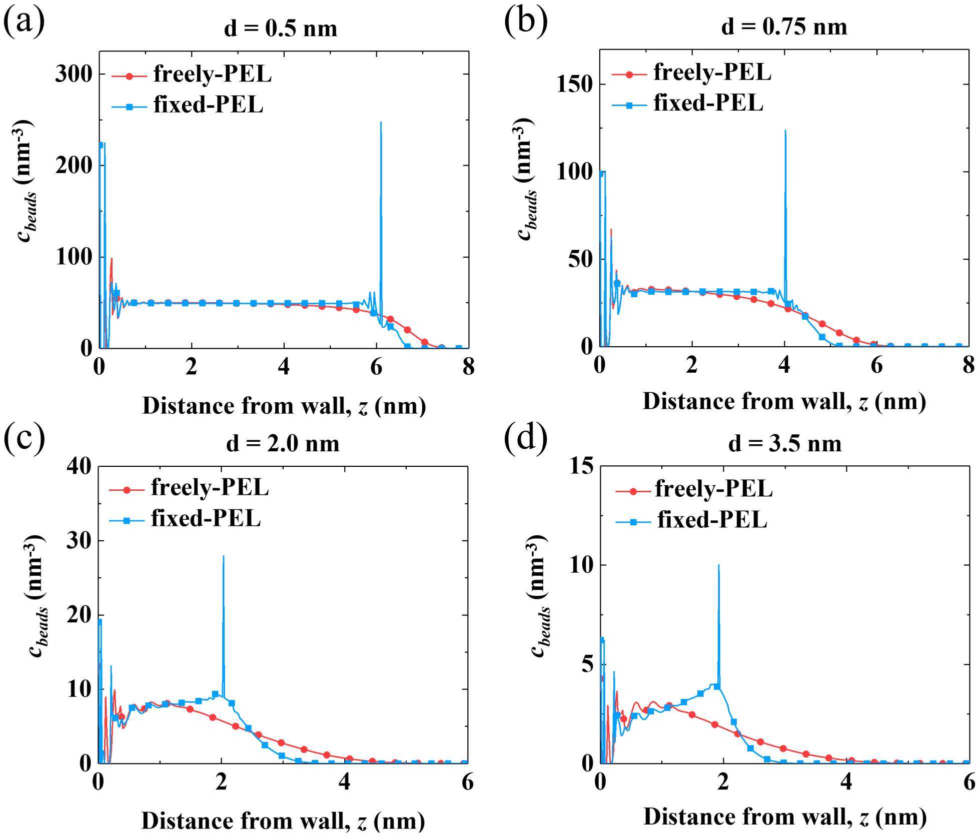

Figure S7: **Comparison of the concentrations of PE beads c_{beads} in the fixed-PELs systems and the freely-PELs systems.** The end-charged PE beads in fixed-PELs system are at the peak position of ρ_{chg} in freely-PELs system.

The effect of space charge density of PE brushes on flow strength

In fact, the charged functional groups are not always located at the tail of PE brushes. We studied the effect of charged functional groups at different locations of PE brushes. The charged polymer beads are distributed at the middle and the tail of PE brushes and such PE brushes are denoted as DC-PE brushes. The flow strengths over DC-PE brushes are compared with that through end-charged PE brushes. The result shows that the flow strength through DC-PE brushes is weaker than that through end-charged PE brushes. Moreover, the difference of flow strength varies with the conformation of PE brushes. For polymers in the brush regime ($d = 0.5, 0.75$ nm), the difference of flow strength is large (Fig. S8(a)). This likely results from a large difference of the net ion concentration between DC-PE and end-charged PE systems (Fig. S8(b)). For polymers in the brush regime, polymers are stretched and the electrostatic interactions from the distributed charges at the middle part of the DC-PE brushes attract ions to distribute closer to the wall, resulting in a large loss of driving effect. The flow strength V_0 increases at concentrated brush regime due to the expulsion of ions from PELs. For PEs in mushroom regime ($d = 2.0, 3.5$ nm), the net concentration of ions between end-charged PE and DC-PE shows similar distribution (Fig. S8(c-d)) and their flow velocities do not differ too much. This likely results from the fact that there is more space available in the mushroom regime and the distribution of polymer beads in the end-charged PE system is similar to that of the charged beads in the DC-PE system. The above observations are in-line with the findings on the effect of distributed charged polymer beads published earlier by Chen and Das[2] and Yeh *et al.*.[3]

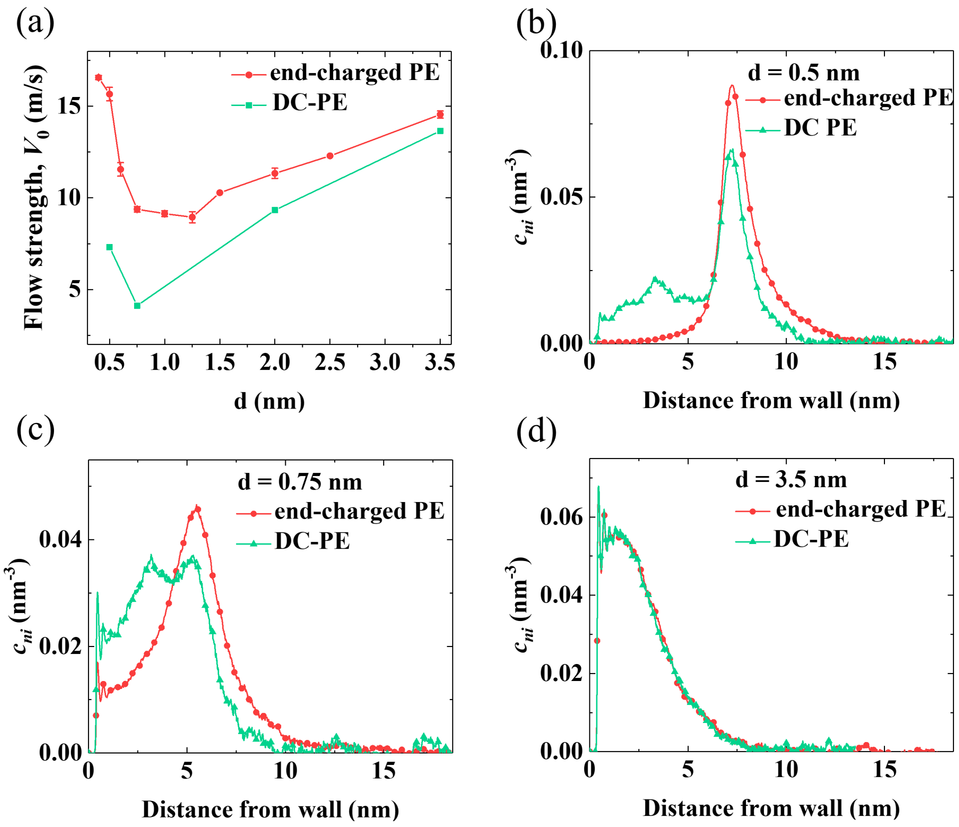

Figure S8: **Effect of space charge density of PE brushes on the variation of flow strength V_0.** (a) Flow strength through end-charged PE brushes and PE brushes with distributed charged functional groups (DC-PE). (b-d) Comparison of net concentration of ions between end-charged PE brushes and DC-PE brushes for separation $d = 0.5, 0.75, 3.5$ nm.

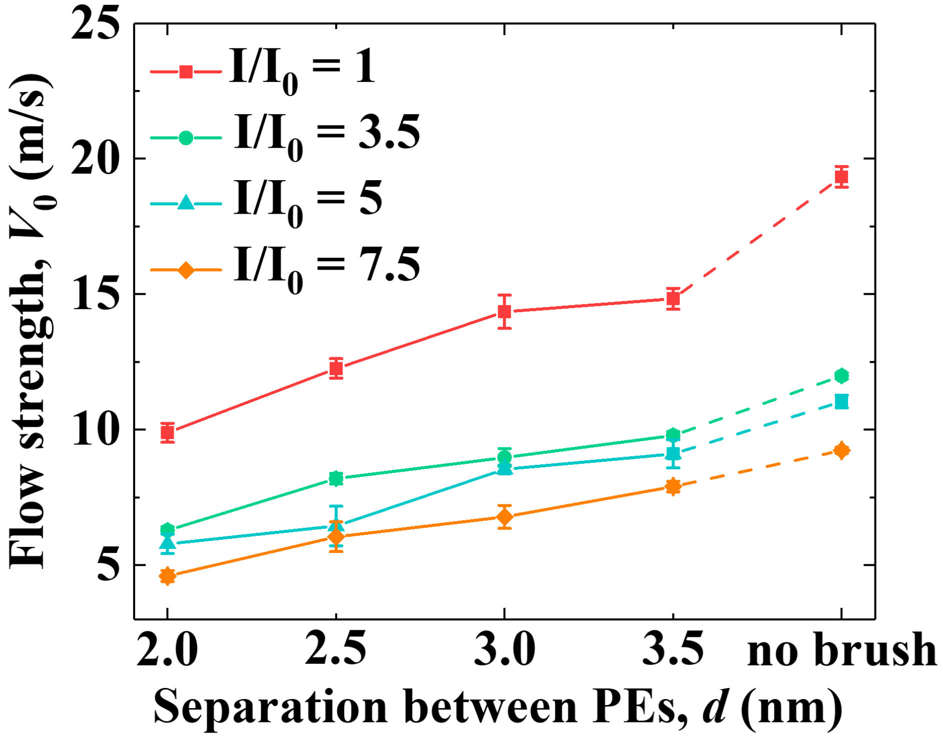

Figure S9: **Effect of ionic strengths on the variation of flow strength V_0.** The solid lines refer to systems grafted with PELs, the dash lines refer to systems with no brushes. The ionic strengths of the system I are normalized by the ionic strength of the baseline system $I_0(I_{bulk})$.

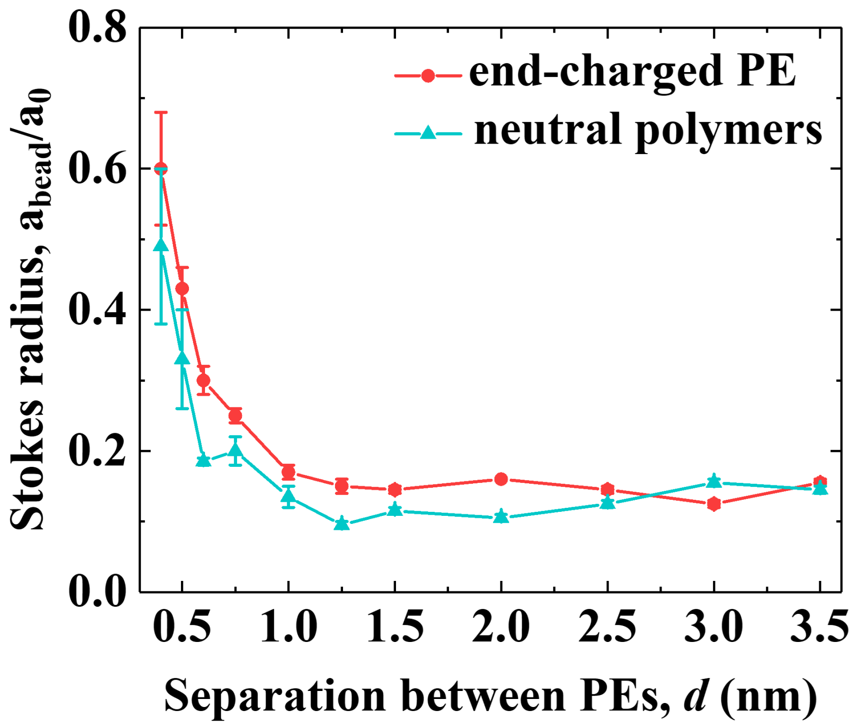

Figure S10: **The Stokes radius of polymer beads a_{bead} as a function of separation between polymers d.** a_{bead} is normalized to the physical size of the beads (*i.e.* Lennard-Jones (LJ) radius, $a_0 = 0.156$ nm). a_{bead} is about one order of magnitude smaller than the physical size of the polymer beads, as demonstrated in our previous work.[4]

Table S1: Parameters of molecular dynamics simulations.

Parameters	Symbol	Value	Unit
Lateral dimension of channel	L_x,L_y	5.0,6.0	nm
Height of channel	H	27,37	nm
Density of wall atoms	ρ_w	33.3	nm^{-3}
Surface charge density	σ_s	3.2×10^{-2}	C/m^2
Degree of polymerization	N	4,8,12,16,20,24	N/A
Density of solvent	ρ_s	49.1	mol/L
Ionic strength of electrolyte	I_{bulk}	3.4×10^{-2}	mol/L
Strength of external electric field	E_{ext}	8×10^{-2}	V/nm
Solvent dielectric constant	ε_s	78	N/A
Viscosity of bulk solvent [a]	μ_s	1.78×10^{-4}	$kg\,m^{-1}\,s^{-1}$
Temperature of system	T	300	K
Pressure of system	P	1	bar
Time step of MD system	ΔT	4	fs

[a] The viscosity of bulk solvent is determined by the method in our previous work Ref.[5]

References

(1) Chen, W. L.; Cordero, R.; Tran, H.; Ober, C. K. 50th anniversary perspective: polymer brushes: novel surfaces for future materials. *Macromolecules* **2017**, *50*, 4089–4113.

(2) Chen, G.; Das, S. Massively Enhanced Electroosmotic Transport in Nanochannels Grafted with End-Charged Polyelectrolyte Brushes. *J. Phys. Chem. B* **2017**, *121*, 3130–3141.

(3) Yeh, L.-H.; Zhang, M.; Qian, S.; Hsu, J.-P.; Tseng, S. Ion Concentration Polarization in Polyelectrolyte-Modified Nanopores. *J. Phys. Chem. C* **2012**, *116*, 8672–8677.

(4) Wu, P.; Sun, T.; Jiang, X.; Kondrat, S. Hydrodynamic Properties of Polymers Screening the Electrokinetic Flow: Insights from a Computational Study. *Polymers* **2019**, *11*, 1038.

(5) Wu, P.; Qiao, R. Physical origins of apparently enhanced viscosity of interfacial fluids in electrokinetic transport. *Phys. Fluids* **2011**, *23*, 072005.
